# Endoscopic surveillance for colorectal cancer and its precursor lesions in Lynch syndrome; time for some policy shifts?

**DOI:** 10.1186/s13053-025-00312-z

**Published:** 2025-04-16

**Authors:** Romy N Kuipers, Marissa F Burggraaff, Michiel HJ Maas, Dorien TJ van der Biessen – van Beek, Mariëtte CA van Kouwen, Tanya M Bisseling

**Affiliations:** Department of Gastroenterology and Hepatology, Radboud University Medical Center, Nijmegen, The Netherlands

**Keywords:** Lynch syndrome, Adenomatous polyps, Colorectal cancer, Endoscopic surveillance, T1 cancer

## Abstract

**Background:**

While numerous studies have demonstrated variations in colorectal cancer (CRC) incidence among Lynch Syndrome (LS)-associated germline pathogenic variant (gPV) carriers, limited data are available regarding tailoring surveillance and treatment strategies. The main goal of this study was to estimate whether personalised care could be offered based on the different gPVs (*MLH1*, *MSH2*, *MSH6* or *PMS2*). Additionally, the outcome from patient-shared care for early (T1) CRC was investigated.

**Methods:**

The study is performed as a single centre retrospective analysis of our cohort of patients with a LS-associated gPV in *MLH1*, *MSH2*, *MSH6* or *PMS2.* Colon surveillance data from between January 1978 to February 2024 were collected. Analyses were performed to identify differences in incidence of precursor lesions and CRC between the different variants and treatment variation for CRC in LS.

**Results:**

From a cohort of 621 LS individuals 496 (133 *MLH1*, 107 *MSH2*, 180 *MSH6* and 76 *PMS2*) could be included in this study. Analyses revealed that, despite adequate surveillance intervals and lower adenoma incidence, individuals with a gPV in *MLH1* or *MSH2* have higher CRC incidences compared to *MSH6* or *PMS2.* Most detected CRC lesions were early stage (T1) CRCs. Treatment for T1 CRC varied considerably; in 68% of the cases deviating from a subtotal colectomy, with nearly equivalent recurrence rates.

**Discussion:**

Based on higher precursor lesion detection and lower CRC incidences in LS individuals with a gPV in *MSH6* or *PMS2* under biannual endoscopic surveillance, this study supports the potential for extended surveillance intervals in the latter group. As treatment for the detected T1 CRCs varied considerably with nearly equivalent recurrence rates, in selected cases less invasive interventions for LS individuals could be considered.

**Supplementary Information:**

The online version contains supplementary material available at 10.1186/s13053-025-00312-z.

## Introduction

Lynch syndrome (LS) is an autosomal dominant cancer susceptibility disorder arising from germline pathogenic variants (gPV) in one of the four mismatch repair (MMR) genes [1]. It is estimated that 1 in 1946 persons carry a gPV in *MLH1*, 1 in 2841 in *MSH2*, 1 in 758 in *MSH6* and 1 in 714 in *PMS2* [2]. Individuals with a gPV in one of the MMR genes face a hereditary increased risk of developing cancer, in particular early-onset colorectal cancer (CRC) and endometrial cancer [3, 4]. The lifetime risk of developing CRC differs per gPV, ranging from 40 to 50% for patients carrying gPVs in *MLH1* or *MSH2* genes to 10–20% in *MSH6* and *PMS2* genes [5, 6].

In an attempt to reduce CRC incidence, all LS patients are offered endoscopic surveillance. Since the detection of the first LS related mutations in the mid- 1990 s, surveillance is regulated in guidelines. In earlier years, surveillance was based on family history and/or the medical doctor’s expert opinion. The aims of endoscopic surveillance are to timely remove precursor lesions that could potentially develop into CRC, as well as to enable the detection of CRC at earlier stage thereby facilitating early intervention [7]. Most current guidelines advise performing surveillance colonoscopies every two years, regardless of mutation type. Although biennial surveillance has led to significant improvements in LS prognosis [8], adherence to timely colonoscopy surveillance is suboptimal [9, 10]. This is most likely due to patients’ perceived barriers regarding colonoscopies such as discomfort and time consumption. Over the years, the detection of precursor lesions has improved, partly due to increased awareness of which lesions to target during endoscopy and partly because of advancements in the quality of endoscopes. With the knowledge that substantial differences in CRC incidence [5, 6] and tumour biology [11] among the different MMR gPVs exist, there is potential for less invasive and more personalised surveillance strategies for certain patient groups. Limited data support the extension of surveillance intervals for carriers of pathogenic variants *MSH6* and *PMS2* [7], who are proven to have lower cumulative CRC incidences, possibly due to this different tumour biology [11]. 

There is ongoing debate about the optimal treatment protocol for patients with LS. Current Dutch guidelines recommend all patients to undergo a subtotal colectomy upon detection of CRC during surveillance [12]. The latest European guideline from the European Hereditary Tumour Group (EHTG) and European Society of Coloproctology (ESCP) suggests that, given the relatively low lifetime risk of developing CRC and metachronous CRC, more bowel-conserving therapies should be considered for *MSH6* and *PMS2* mutation carriers, such as partial colectomies [5]. In daily practice, variability exists ranging from performing partial colectomies to even local endoscopic resections. The latter possibly due to the increasingly common local treatment of sporadic early (T1) CRC [13]. Uncertainty surrounds the optimal approach for treating T1 CRC in LS patients, in particular whether local treatment or surgical intervention is preferable, and which option yields the lowest recurrence rates [13, 14]. This uncertainty arises partly from the fact that tumour biology in LS differs from sporadic CRC. Recently in *MLH1* a non-adenoma pathway that leads to CRC has been described [15]. It is unsure if treatment options for a sporadic T1 CRC can safely be copy-pasted on LS related T1 CRC.

Over the last 5 to 10 years, two major data cohorts for LS have been developed. One cohort originates from the InSIGHT database (https://insight-database.org/), which estimates cancer risks based on specific genetic variants. Additionally, the Prospective Lynch Syndrome Database (PLSD, https://plsd.eu/) was recently developed. This database includes surveillance data from about 6000 LS carriers, providing cancer incidence statistics categorized by affected gene, gender and age. Both databases are populated by individual caretakers, without verification for accuracy or completeness. Moreover, the data come from numerous hospitals, often with only a small number of cases per institution, which introduces the risk for (unknown) treatment variation. Both InSIGHT and PLSD do not report advanced neoplasia, second and third CRCs and treatment (variation). Understanding whether specific Lynch-associated gPVs exhibit differential detection rates of advanced neoplasia and 2nd/3rd CRC as well as evaluating existing treatment modalities can be of additional value for tailoring surveillance and treatment strategies.

The aim of this single centre cohort analysis is to evaluate both the occurrence and management of CRC and its precursor lesions during surveillance across patients with LS with different MMR-gene gPVs. The findings of this study may contribute to shape risk stratification, surveillance- and treatment protocols, ultimately leading to more personalized interventions and improved outcomes for LS patients.

## Methods

### Study design

This retrospective cohort analysis was conducted at the Radboud university medical centre in Nijmegen, the Netherlands. Patients that underwent surveillance colonoscopies for LS between January 1978 and February 2024 were screened for eligibility. Patients that were eligible to be included in the study were stratified into four groups according to their gPV status. The study was ethically approved by the ‘Central Committee on Research involving Human subjects’ dossier number ‘2023–16812’.

### Study population

Every patient who carried one of the LS-associated gPVs (*MLH1*, *MSH2*, *MSH6* or *PMS2*) and underwent at least one surveillance colonoscopy after their index colonoscopy between January 1978 and February 2024 could be included. Patients were excluded once they carried multiple LS-associated gPVs, received an experimental LS vaccination or underwent colectomy without available information regarding the procedure.

### Data collection

Baseline characteristics such as age, gender and gPV type were extracted from electronic health records. In addition, information regarding the performed surveillance colonoscopies, corresponding pathology reports and performed treatment was gathered. All data were checked by two researchers (MB and TB).

### Outcomes

The primary outcome consisted of incidences of precursor lesions and CRC among the different gPVs during the surveillance period. Precursor lesions were defined as non-advanced adenoma of < 10 mm and advanced adenoma of ≥ 10 mm. In addition, the various treatment modalities for LS related early stage (T1) CRC were evaluated.

Secondary outcomes involved variations among gPV groups in TNM-staging of the identified CRCs and anatomical locations of precursor lesions.

### Statistical analysis

In this study, a *p*-value of < 0.05 was considered statistically significant and 95% confidence intervals (CI) were presented when appropriate. Data analyses were performed with the use of IBM SPSS Statistics for Windows, version 29.0. All data were reported as mean with standard deviation (SD) or median with interquartile range (IQR). Categorical outcomes were presented as counts with percentages. Differences between mutation variants were analysed using Chi-square, Fisher-Freeman-Halton exact, one way ANOVA or Kruskal Wallis tests. Kaplan Meier time-to-event analyses with log-rank tests were performed to calculate the cumulative incidence of CRC, after which Cox regression analyses with corresponding hazard ratios (HR) and 95% CIs were performed to evaluate variations between the mutation groups.

## Results

**Fig. 1:**
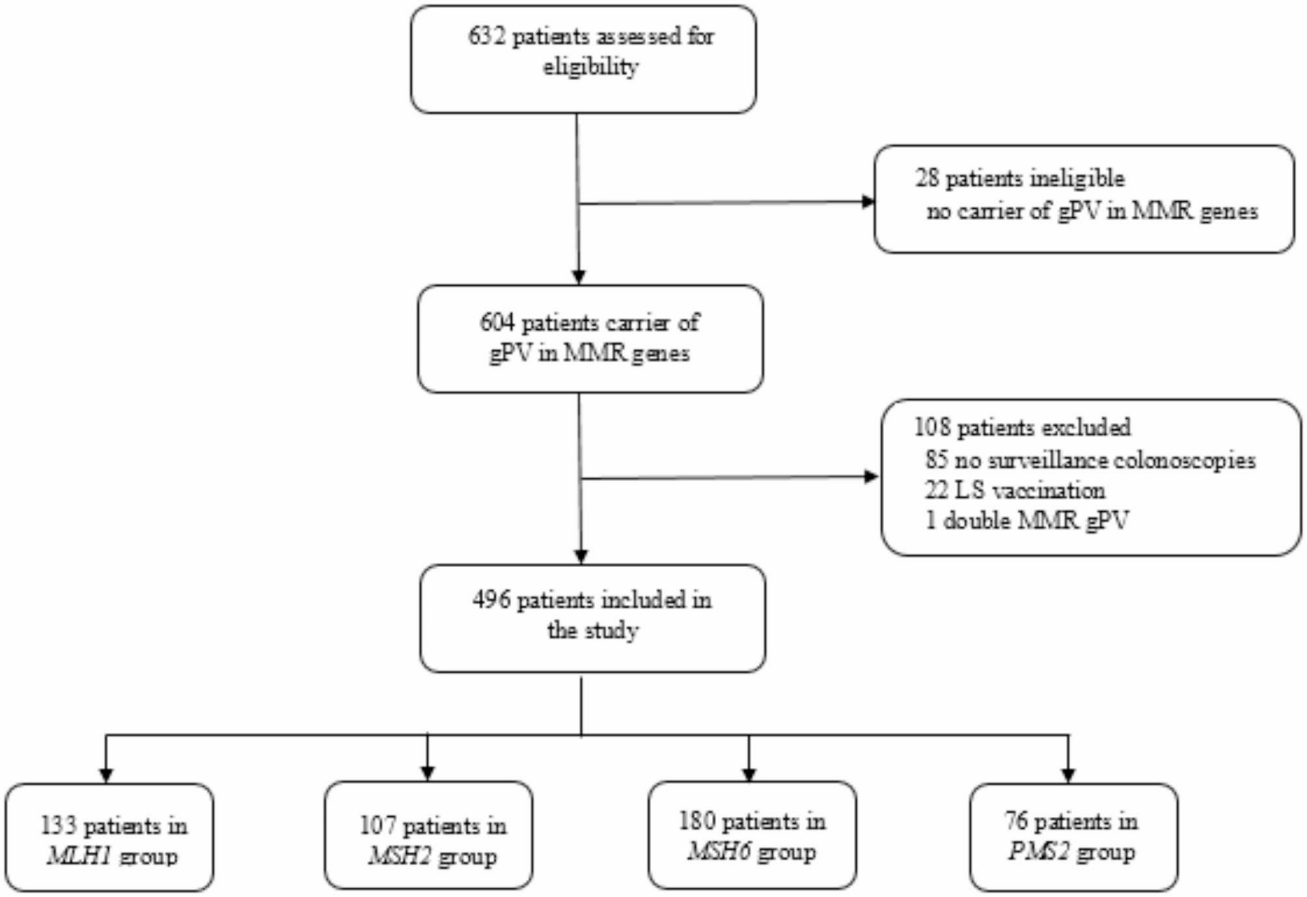
Flowchart of inclusion

In total 632 LS individuals were assessed for eligibility of which 604 individuals were confirmed carriers of gPVs in one of the MMR genes. Of those, 108 matched exclusion criteria ultimately leading to the inclusion of 496 LS individuals; 133 in the *MLH1* group, 107 in the *MSH2* group, 180 in the *MSH6* group and 76 in the *PMS2* group (Fig. 1).

The baseline characteristics are presented in Table 1. Individuals carrying *MLH1* or *MSH2* gPVs were younger at the time of LS diagnosis compared to those carrying *MSH6* and *PMS2* gPVs, with median ages of 34 and 35 versus 41 and 48 years (*p* < 0.001), respectively. The surveillance period for individuals in the *MLH1* and *MSH2* group was longer than for those in the *MSH6* and *PMS2* group (10 and 15 years vs. 7 and 6 years; *p* < 0.001). Additionally, more surveillance coloscopies were performed in the *MLH1* and *MSH2* groups (8 and 8 vs. 5 and 4; *p* < 0.001), with shorter mean intervals between the procedures (1.7 and 1.9 years vs. 2.0 and 2.0 years; *p* = 0.007). A significantly higher incidence of CRC was found during initial colonoscopy in the *MLH1* group (17%) compared to the *MSH2* (8%), *MSH6* (5%) and *PMS2* groups (7%; *p* = 0.004). No differences in detection of precursor lesions during initial colonoscopy were observed across the groups.

**Table 1:** Baseline characteristics

	MLH1 (*n* = 133)	MSH2 (*n* = 107)	MSH6 (*n* = 180)	PMS2 (*n* = 76)	*P*-value
Sex (male)	61 (46%)	50 (47%)	71 (39%)	35 (46%)	0.542
Age (years)	52 (37–63)	50 (40–62)	53 (42–66)	55 (46–68)	0.036^*^
Age LS diagnosis	34 (26–47)	35 (27–48)	41 (33–54)	48 (39–55)	< 0.001^*^
Age first CRC	46 (12)	46 (13)	47 (12)	45 (13)	0.963
Lesions found during initial colonoscopy					
Non-advanced adenomas	23 (17%)	18 (17%)	39 (22%)	14 (18%)	0.698
Advanced adenomas	3 (2%)	3 (3%)	6 (3%)	2 (3%)	0.980
CRC	22 (17%)	9 (8%)	9 (5%)	5 (7%)	0.004^*^
Total surveillance period (years)	10 (5–19)	15 (5–19)	7 (4–13)	6 (2–10)	< 0.001^*^
Performed colonoscopies	8 (4–13)	8 (4–13)	5 (3–8)	4 (2–6)	< 0.001^*^
Surveillance interval (years)	1.7 (1.3–2.2)	1.9 (1.5–2.2)	2.0 (1.7–2.2)	2.0 (1.8–2.3)	0.007^*^

Data are mean (SD) or median (IQR) and n (%). *LS* Lynch syndrome, *CRC* colorectal cancer. Advanced adenomas are defined as adenomas > 10 mm or with high-grade dysplasia. Non-advanced adenomas are adenomas < 10 mm and without high-grade dysplasia

^*^Significant with *p*<0.05

**Fig. 2:**
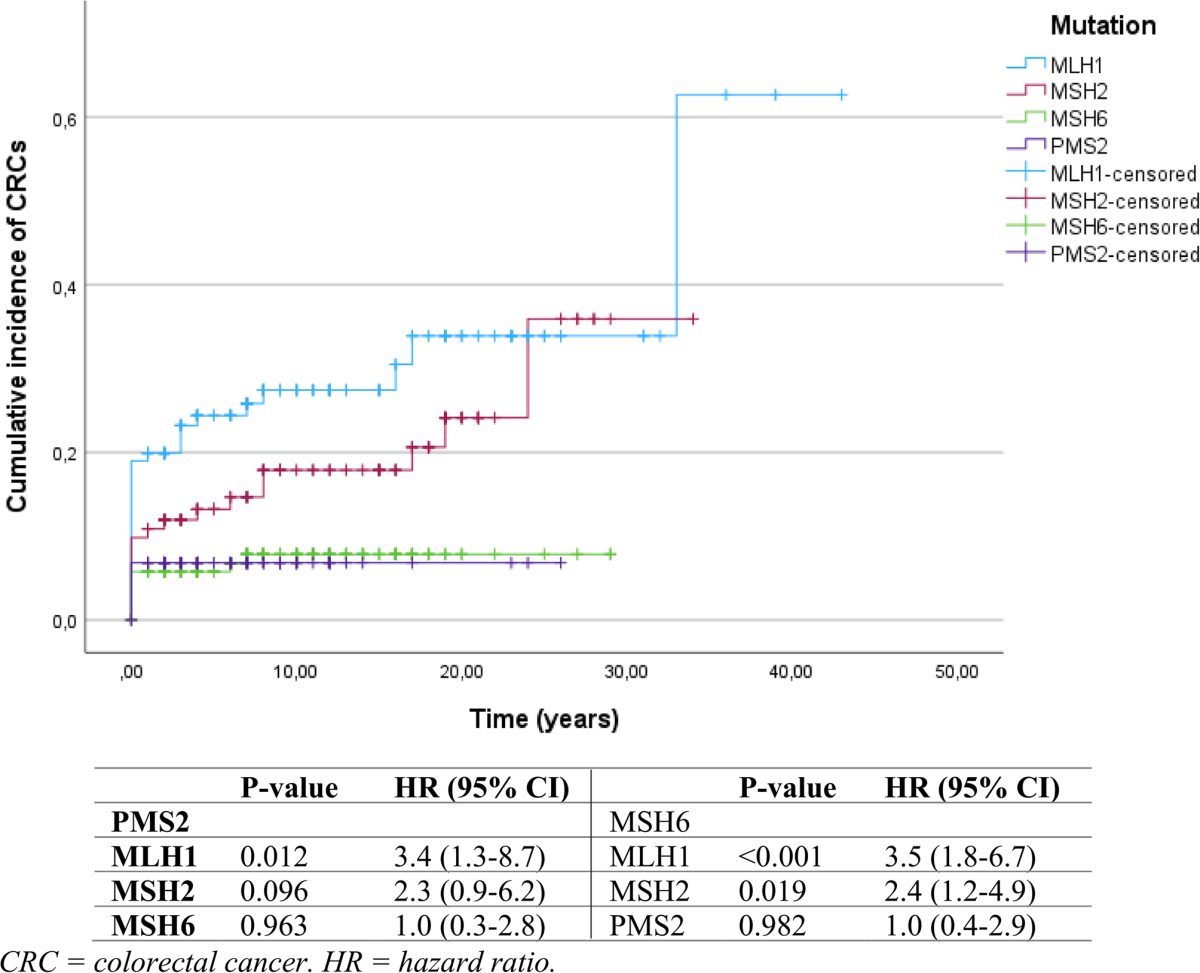
Cumulative life-time incidence of CRC per mutation group

Individuals carrying gPVs in *MLH1* and *MSH2* showed significantly higher cumulative lifetime incidences of CRC compared to those with *MSH6* gPVs (*p* < 0.001; *p* = 0.021; Fig. 2). Within the *MLH1* group, more CRCs were detected in men (*n* = 16/61, 26%) compared to women (*n* = 6/72, 8%). Additionally, individuals with a *MLH1* gPV showed significantly higher cumulative lifetime incidences of CRC compared to *PMS2* gPV carriers (*p* = 0.012). In terms of lifetime CRC risks, individuals in both the *MLH1* and *MSH2* groups exhibited higher lifetime risks compared to individuals in the *MSH6* group, with HRs of 3.5 (95% CI 1.8–6.7) and 2.4 (95% CI 1.2–4.9), respectively. Individuals in the *MLH1* group also showed a significantly higher lifetime CRC risk compared to those in the *PMS2* group (HR 3.4; 95% CI 1.3–8.7).

Eighteen individuals with LS caused by a gPV in one of the MMR genes developed two CRCs outside the surveillance setting. Seven of these cases involved synchronous cancers (1 *MLH1*, 4 *MSH2*, 2 *MSH6*). Eleven individuals had their first CRC in the 1980 s or early 1990 s, with a LS gPV identified only after the detection of the second CRC. None of these individuals developed a third CRC during subsequent surveillance.

The most frequently detected precursor lesions throughout the surveillance period were non-advanced adenomas (89%). The incidence of non-advanced adenomas was significantly higher in individuals with *MSH6* (96%) and *PMS2* (94%) variants compared to those with *MLH1* (87%) and *MSH2* (84%) variants (*p* = 0.001; Table 2). CRC was identified in 5% of the individuals with LS during the surveillance period. When stratified by mutation type, individuals with a *MLH1* or *MSH2* gPV developed CRC more frequently, with incidences of 8% and 7%, respectively. In contrast, only 2% of individuals with *MSH6* or *PMS2* gPV developed CRC. The majority of CRCs were located right-sided (Table 2).

**Table 2:** Surveillance colonoscopies

	Total (*n* = 496)	MLH1 (*n* = 133)	MSH2 (*n* = 107)	MSH6 (*n* = 180)	PMS2 (*n* = 76)	*P*-value
Lesions detected during surveillance						
Non-advanced adenoma	569 (89%)	167 (87%)	177 (84%)	173 (96%)	50 (94%)	0.001^*^
Advanced adenoma	69 (11%)	21 (11%)	29 (14%)	13 (7%)	6 (11%)	0.225
Colorectal carcinoma	32 (5%)	15 (8%)	14 (7%)	3 (2%)	1 (2%)	0.024^*^
Location of non-advanced adenoma						0.518
Right-sided	306 (54%)	98 (59%)	87 (49%)	89 (51%)	31 (62%)	
Left-sided	194 (34%)	52 (31%)	67 (38%)	62 (36%)	13 (26%)	
Right and Left- sided	68 (12%)	17 (10%)	23 (13%)	22 (13%)	6 (12%)	
Location of advanced adenomas						0.089
Right-sided	38 (55%)	12 (57%)	12 (41%)	9 (69%)	5 (83%)	
Left-sided	30 (44%)	9 (43%)	17 (59%)	3 (23%)	1 (17%)	
Right and Left- sided	1 (1%)	0 (0%)	0 (0%)	1 (8%)	0	
Location of CRC						0.355
Right-sided	23 (70%)	12 (80%)	9 (64%)	2 (67%)	0	
Left-sided	9 (30%)	3 (20%)	5 (36%)	1 (33%)	1 (100%)	

Data are median (IQR) and n (%). *CRC* colorectal cancer. Advanced adenomas are defined as adenomas > 10 mm or with high-grade dysplasia. Non-advanced adenomas are adenomas < 10 mm and without high-grade dysplasia. Lesions are considered right-sided when located proximal of the splenic flexure. Lesions are considered left sided when located distal of the splenic flexure

^*^Significant with *p*<0.05

As shown in Table 3, the majority of CRCs identified during surveillance were classified as T1 tumours (52%), with none classified as T4 tumours. The vast majority did not exhibit lymph node (85%) or distant metastases (97%). No significant differences in TNM stages were found among the various gPV groups. Among individuals with LS who developed T1 CRC during their lifetime, 32% underwent subtotal colectomy, while 32% and 36% received partial colectomy and local treatment (Table 4). Among those with T1 CRC detected during surveillance, where treatment deviated from the applicable guideline, one individual was diagnosed with a second carcinoma. This patient had a T1 rectal carcinoma during the index colonoscopy, which was treated by low anterior resection. The second CRC was identified in the ascending colon 12 years later. No metastases were detected and this CRC was adequately resected by right hemicolectomy. Over the subsequent 13 years of follow up, no third CRC has been detected in this individual.

**Table 3:** TNM stadia of detected CRCs

	Total (*n* = 78)	MLH1 (*n* = 37)	MSH2 (*n* = 23)	MSH6 (*n* = 12)	PMS2 (*n* = 6)	*P*-value
Tumor						0.151
Intramucosal	1 (3%)	0	0	1 (8%)	0	
T1 (%)	25 (32%)	10 (27%)	12 (52%)	1 (8%)	2 (33%)	
T2 (%)	19 (24%)	11 (30%)	4 (17%)	3 (25%)	1 (17%)	
T3 (%)	27 (35%)	11 (30%)	7 (30%)	7 (58%)	2 (33%)	
T4 (%)	3 (4%)	3 (8%)	0	0	0	
Node						0.283
N0 (%)	57 (73%)	29 (78%)	18 (78%)	7 (58%)	3 (50%)	
N1 (%)	11 (14%)	3 (8%)	4 (17%)	3 (25%)	1 (17%)	
N2 (%)	7 (9%)	3 (8%)	1 (4%)	2 (17%)	1 (17%)	
Metastasis						0.122
M0 (%)	74 (95%)	35 (95%)	23 (100%)	11 (92%)	5 (83%)	
M1 (%)	1 (1%)	0	0	1 (8%)	0	

**Table 4:** Incidences of performed treatments for T1 CRC removal in Lynch patients

	Total (*n* = 25)	MLH1 (*n* = 10)	MSH2 (*n* = 12)	MSH6 (*n* = 1)	PMS2 (*n* = 2)	*P*-value
						0.945
Subtotal colectomy	8 (32%)	4 (40%)	4 (33%)	0	0	
Partial colectomy	8 (32%)	3 (30%)	3 (25%)	1 (100%)	1 (50%)	
Local endoscopic treatment	9 (36%)	3 (30%)	5 (42%)	0	1 (50%)	

Data are n (%). *CRC* colorectal cancer

## Discussion

Numerous studies demonstrated variations in CRC incidence among different LS-associated genes, yet limited knowledge exists regarding the customisation of surveillance and treatment strategies based on these variations. Therefore, our observational cohort study aimed to sort variations in occurrence and management of CRC and its precursor lesions during surveillance across different LS gPV groups. In line with current knowledge [16], we found that individuals with a gPV in *MLH1* and *MSH2* have higher life-time risks to develop CRC compared to those with *MSH6*. Specifically, the CRC risk is higher in men than women. Furthermore, we confirmed that CRC in individuals with *MLH1* and *MSH2* gPVs often develops without visible precursor lesions, whereas in individuals with *MSH6* and *PMS2 gPVs*, significantly more visible precursor lesions are detected. Additionally, we observed considerable variation in performed treatments for T1 CRC.

This study possesses several strengths. To our knowledge, it represents one of the largest single-center LS surveillance cohorts with complete surveillance data. It reports CRC detection rates during surveillance across four different LS-associated gPVs over a span of four decades. Additionally, beyond CRC incidence, the study also analyses the incidence of precursor lesions, metachronous CRCs, TNM stages for all CRCs and treatment strategies. In contrast to prior research, this study exclusively included individuals with confirmed LS-associated gPVs. All participants were under surveillance at a specialised LS centre staffed with experienced endoscopists. Lastly, to guarantee optimal quality, all data were double-checked, minimizing the risk of information bias.

Regarding the big data databases, such as InSIGHT and PLSD; these are valuable for identifying patterns and are therefore possibly useful as predictive models. These databases include a tenfold larger number of LS carriers compared to our single-center cohort. However, there are several limitations to the PLSD database. First, the distribution of the four MMR genes is skewed with *MSH6* and *PMS2 gPVs* in the minority. Because of this data for individuals with *MSH6* and *PMS2 gPVs* should be interpreted with caution. Additionally, the data from these large databases come from multiple hospitals, each including a significantly lower number of LS carriers compared to our cohort. Furthermore, InSIGHT and PLSD data are reported by consulting specialists without double-checking, which may introduce selection- and reporting bias. Lastly, these databases do not include data on precursor lesions and treatment variations.

This study also exhibits several limitations. The retrospective design unavoidably resulted in missing data. However, despite the amount of missing data, significant differences or trends were still observed. Second, the timing of precursor and carcinoma detection relied on the timing of colonoscopy procedures, which may have introduced information bias. In addition, improvements in imaging quality over time may have influenced lesion detection, potentially resulting in a higher number of lesions being detected in patients under surveillance nowadays compared to those monitored in the past. Though, in our cohort, 69% of individuals with LS have been under surveillance during the last 15 years, indicating that the majority of individuals with LS have been monitored with the most modern endoscopic equipment. Both the timing of colonoscopy procedures and improvements in imaging quality may have influenced the overall detection rate, but these limitations are not expected to have directly impacted the proportion of detected lesions among mutations groups, as all patients underwent similar surveillance protocols. Lastly, a limitation of our single-centre design is that the generalizability of these findings to the broader global LS population may be questioned.

Performing biennial surveillance colonoscopies in individuals with LS has been shown to be essential, as it substantially improved the prognosis of individuals with LS caused by a gPV in one of the MMR genes, due to earlier detection of CRC and premalignant adenomas [8]. However, the effectiveness of the surveillance program heavily relies on adherence, and several studies already demonstrated adherence to timely colonoscopy surveillance in individuals with LS is not optimal [9, 10]. In a study of Van Liere et al. [17], it was shown that 57% of individuals with LS perceived the surveillance program as extremely burdensome, with 10% even considering quitting surveillance. Many individuals (60%) preferred a less invasive surveillance method. The current biennial surveillance program, therefore, appears to negatively influence quality of life, particularly if biennial checks may not even be necessary for certain LS associated gPV groups.

Earlier studies have already demonstrated variability in the lifetime risks of developing CRC among different individuals with LS, with carriers of a gPV in *MLH1* and *MSH2* being at the highest hereditary CRC risk [16]. Consistently, in this study, we observed that individuals with *MLH1* gPV exhibited significantly higher CRC incidences at initial colonoscopy compared to other LS-associated gPV groups (Table 1). In addition, with regard to detected lesions during surveillance colonoscopies, higher CRC incidences were observed in the *MLH1* and *MSH2* compared to the *MSH6* and *PMS2* gPV groups. At the same time, the observed non-advanced adenoma incidence was lower in *MLH1* and *MSH2* gPV groups. This findings aligns with recent insights into CRC pathways in LS, which suggest three different pathways [15]. It is proposed that CRC development in individuals with *MLH1* and *MSH2* gPVs follows a more accelerated pathway with MMR deficient precursor foci in the colonic crypts [18]. In individuals with a *MLH1* gPV, these foci may be located beneath the mucosal layer making them easily overlooked [18]. Thus, since the tumour biology of a *MLH1* gPV differs from that of an *MSH2* gPV, they cannot be considered as an equal entity.

The observed cumulative lifetime CRC incidence was higher in the *MLH1* compared to *MSH6* and *PMS2* variant groups, as well as in *MSH2* compared to the *MSH6* variant group (Fig. 2). These findings suggest that, given the substantially lower lifetime risk of developing CRC and considering the negative impact of biennial surveillance on quality of life, surveillance intervals for individuals with *MSH6* and *PMS2* gPVs could potentially be safely extended.

We additionally analysed differences in TNM staging across the different gPV groups, as well as the corresponding treatments for early-stage (T1) CRC. As expected from the implementation of biennial surveillance, most CRCs detected during surveillance colonoscopies were T1 CRCs (52%) without the presence of lymph node- (85%) and distant (97%) metastasis (Table 3). When examining the relatively small number of patients with treated early-stage colorectal T1 CRCs (*n* = 25), we observed considerable variation in the performed surgical or endoscopic treatment (Table 4). In 68% of the cases, physicians deviated from the applicable guideline, which recommended subtotal colectomy for early stage (T1) CRC [12]. Less invasive local endoscopic treatments were performed more frequently than the resection advised by the current national guideline (36% vs. 32%), clearly demonstrating preference and confidence in more local methods of treatment. Given the significant advancement in endoscopic techniques [19, 20] and improved functional outcome after more local treatments [21], it is not surprising there has been a growing preference for minimally invasive interventions in recent years. The current guideline for non-LS related CRC even already includes more tailored treatment recommendations for T1 tumours (https://richtlijnendatabase.nl/richtlijn/colorectaal_carcinoom_crc/startpagina_-_colorectaal_carcinoom.html#:~:text=De%20richtlijn%20colorectaal%20carcinoom%20richt,bij%20(verdenking%20op)%20darmkanker), with endoscopic techniques considered superior to surgical resection in most cases. Specific histological risk factors for non-LS related colorectal T1 CRCs, including poor differentiation, lymph angioinvasion and high-grade tumour budding, have been identified as predictors of metastasis and recurrence [22]. After endoscopic removal of non-LS related colorectal T1 CRCs, the presence or absence of these histological risk factors can subsequently assist in determining the need for additional surgical resection and/or in formulating the appropriate surveillance strategy [22]. Interestingly, only one of the LS patients treated for T1-stage CRC experienced a second CRC twelve years after a CRC was detected during the index colonoscopy, suggesting less invasive organ preserving treatment for LS-related colorectal T1 CRCs might be a viable option. Nonetheless, evaluation of recurrence rates in the long term is necessary given that local treatments were conducted more recently, leaving the possibility for recurrences to emerge.

In our opinion, future studies should focus on distinguishing more personalised surveillance intervals for different LS associated gPV groups, possibly enabling extension of surveillance intervals for individuals with *MSH6* and/or *PMS2* gPVs given the smaller lifetime risks of developing CRC. Regarding treatment strategies for colorectal T1 CRCs, it is crucial to validate these findings in larger patient cohorts, with particular emphasis on monitoring the long-term outcomes of endoscopic treatments. In addition, it is interesting to investigate whether the current histological risk factors for non-LS related colorectal T1 CRCs also apply to LS-related colorectal T1 CRCs, and if so, whether these risk factors differ across the various LS associated gPV groups.

In conclusion, this large single centre analysis showed a higher precursor lesion detection rate as well as a lower CRC incidence for individuals with LS caused by a gPV in *MSH6* or *PMS2*, suggesting the potential for extended surveillance intervals for these patients. Furthermore, consistent with biennial surveillance protocols, the majority of CRCs detected during surveillance were T1 CRCs without metastases. Regarding treatment of these T1 CRCs, considerable variation was observed with nearly equivalent recurrence rates, highlighting the feasibility of less invasive endoscopic interventions for individuals with LS. To further refine surveillance strategies, additional research into extending surveillance intervals for individuals with LS caused by a gPV in *MSH6* and *PMS2* is needed. Moreover, assessing the long-term outcomes of diverse treatment modalities and identifying histological risk factors specific to LS-related colorectal T1 CRCs are essential steps towards optimising future treatment for individuals with LS.

## Supplementary Information

Supplementary Material 1.

## Data Availability

No datasets were generated or analysed during the current study.
