# Supplementary material for: Endoscopic surveillance for colorectal cancer and its precursor lesions in Lynch syndrome; time for some policy shifts?
Source: Hered Cancer Clin Pract. 2025 Apr 16;23:13. doi: 10.1186/s13053-025-00312-z (PMC12001557; doi:10.1186/s13053-025-00312-z)
Supplement: Supplementary file 1 — Supplementary Material 1. [file 13053_2025_312_MOESM1_ESM.docx]

**Supplementary table 1** Baseline characteristics per gender

|  | MLH1 (n = 133) |  | MSH2 (n = 107) |  | MSH6 (n = 180) |  | PMS2 (n = 76) |  | P-value |
| --- | --- | --- | --- | --- | --- | --- | --- | --- | --- |
|  | **male** | **female** | **male** | **female** | **male** | **female** | **male** | **female** |  |
| Number | 61 (46%) | 72 (54%) | 50 (47%) | 57 (53%) | 71 (39%) | 109 (61%) | 35 (46%) | 41 (54%) | 0.542 |
| Age (years) | 54 (41-63) | 50 (34-63) | 49 (38-62) | 53 (41-63) | 51 (41-63) | 54 (44-67) | 56 (49-69) | 55 (44-68) | 0.056 |
| Age LS diagnosis | 36 (28-48) | 32 (25-47) | 35 (28-45) | 34 (26-48) | 39 (32-53) | 43 (33-55) | 49 (39-57) | 46 (40-55) | <0.001* |
| Age first CRC | 44 (11) | 48 (12) | 46 (11) | 46 (14) | 50 (9) | 46 (14) | 39 (9) | 60 (11) | 0.598 |
| Lesions found during initial colonoscopy |  |  |  |  |  |  |  |  |  |
| Non-advanced  adenomas | 10 (16%) | 13 (18%) | 9 (18%) | 9 (16%) | 22 (31%) | 17 (16%) | 11 (31%) | 3 (7%) | 0.032* |
| Advanced  adenomas | 1 (2%) | 2 (3%) | 1 (2%) | 2 (4%) | 3 (4%) | 3 (3%) | 2 (6%) | 0 (0%) | 0.877 |
| Colorectal  carcinomas | 16 (26%) | 6 (8%) | 3 (6%) | 6 (11%) | 3 (4%) | 6 (6%) | 3 (9%) | 2 (5%) | <0.001* |
| Total surveillance period (years) | 12 (6-22) | 8 (4-17) | 11 (4-19) | 15 (7-20) | 6 (3-11) | 8 (4-14) | 6 (2-10) | 6 (3-10) | <0.001* |
| Performed   colonoscopies | 9 (5-16) | 7 (3-10) | 7 (3-12) | 9 (4-14) | 5 (3-8) | 5 (3-8) | 5 (2-6) | 4 (3-6) | <0.001* |
| Surveillance  interval (years) | 1.6 (1.2-2.0) | 1.9 (1.4-2.2) | 1.9 (1.5-2.2) | 1.8 (1.4-2.2) | 1.9 (1.4-2.1) | 2.0 (1.7-2.2) | 2.0 (1.9-2.2) | 2.0 (1.7-2.3) | <0.001* |

*Data are mean (SD) or median (IQR) and n (%). LS = Lynch syndrome. CRC = colorectal cancer. Advanced adenomas are defined as adenomas >10mm or with high-grade dysplasia. Non-advanced adenomas are adenomas <10mm and without high-grade dysplasia.*
